# Press water from the mechanical drying of Douglas-fir wood chips has multiple beneficial effects on lignocellulolytic fungi

**DOI:** 10.1186/s40694-022-00141-y

**Published:** 2022-05-23

**Authors:** Manfred J. Reppke, Rebecca Gerstner, Elisabeth Windeisen-Holzhauser, Klaus Richter, J. Philipp Benz

**Affiliations:** 1 Professorship of Fungal Biotechnology in Wood Science, Holzforschung München (HFM), TUM School of Life Sciences, Technical University of Munich, Hans-Carl-von-Carlowitz-Platz 2, 85354 Freising, Germany; 2 Chair of Wood Science, Holzforschung München (HFM), TUM School of Life Sciences, Technical University of Munich, Winzererstr. 45, 80797 Munich, Germany; 3 Institute for Advanced Study, Technical University of Munich, Lichtenbergstraße 2a, 85748 Garching, Germany

**Keywords:** Wood press water, Media supplements, Biorefinery, Lignocellulolytic fungi, *Trichoderma reesei*, Cellulase, Bio-clarification

## Abstract

**Background:**

The mechanical drying of wood chips is an innovative method that improves the heating value of sawmill by-products in an energy-efficient continuous process. The liquid that comes out of the wood chips as press water (PW), however, contains a variety of undissolved as well as dissolved organic substances. The disposal of the PW as wastewater would generate additional costs due to its high organic load, offsetting the benefits in energy costs associated with the enhanced heating value of the wood chips. Our research explored if the organic load in PW could be utilized as a substrate by cellulolytic filamentous fungi. Hence, using the industrially relevant Ascomycete *Trichoderma reesei* RUT-C30 as well as several Basidiomycete wood-rotting fungi, we examined the potential of press water obtained from Douglas-fir wood chips to be used in the growth and enzyme production media.

**Results:**

The addition of PW supernatant to liquid cultures of *T. reesei* RUT-C30 resulted in a significant enhancement of the endoglucanase and endoxylanase activities with a substantially shortened lag-phase. A partial replacement of Ca^2+^, Mg^2+^, K^+^, as well as a complete replacement of Fe^2+^, Mn^2+^, Zn^2+^ by supplementing PW of the liquid media was achieved without negative effects on enzyme production. Concentrations of PW above 50% showed no adverse effects regarding the achievable endoglucanase activity but affected the endoxylanase activity to some extent. Exploring the enhancing potential of several individual PW components after chemical analysis revealed that the observed lag-phase reduction of *T. reesei* RUT-C30 was not caused by the dissolved sugars and ions, nor the wood particles in the PW sediment, suggesting that other, so far non-identified, compounds are responsible. However, also the growth rate of several basidiomycetes was significantly enhanced by the supplementation of raw PW to the agar medium. Moreover, their cultivation in liquid cultures reduced the turbidity of the PW substantially.

**Conclusions:**

PW was identified as a suitable media supplement for lignocellulolytic fungi, including the cellulase and xylanase producer *T. reesei* RUT-C30 and several wood-degrading basidiomycetes. The possibility to replace several minerals, trace elements and an equal volume of fresh water in liquid media with PW and the ability of fungal mycelia to filter out the suspended solids is a promising way to combine biological wastewater treatment with value-adding biotechnological applications.

**Supplementary Information:**

The online version contains supplementary material available at 10.1186/s40694-022-00141-y.

## Introduction

Sawmills generate large quantities of by-products, such as bark, sawdust, and wood chips. The average yield of sawn timber from round wood in sawmills has been reported to be around 60%, which means around 40% of the round wood ends up as a by-product [1–3]. The total production of wood chips, particles and residues in Germany reached an estimated 10.97 million m^3^ in 2019 [4]. Wood chips are often sold to thermal power stations or to the pulp and paper industry. Nonetheless, a large portion of these by-products is generally used for internal energy production [2]. With average moisture contents of 40–50%-wet basis after production, microbial activity is facilitated during storage. This can increase the risk for spontaneous combustion due to heat accumulation inside the piles [5] as well as energy loss due to decreased combustible mater [6–8]. Technical drying is therefore necessary to achieve suitable moisture contents that both prevent microbial activity and guarantee high fuel quality [9, 10].

Conventional drying methods based on the evaporation of moisture mostly use thermal energy from dedicated combustion of biogenic or fossil fuels [11, 12]. Regardless of the type of thermal dryer, energy efficiency and drying rate are critical issues associated with thermal drying methods [13]. Mechanical dewatering, on the other hand, is a process that uses high pressure to force the moisture out [14]. The increased pressure within the wood will force the free water out of the cell lumen [15, 16]. The energy required in compression and thus for expulsion of the water is much lower than the energy required to vaporize the same water using thermal energy [17]. The combination of mechanical squeezing and thermal drying can therefore lead to substantial energy savings compared exclusively thermal drying [18, 19].

Large quantities of press water (PW) are released during the mechanical dewatering of wood chips. Since the PW originates from the free water in the lumen of the wood cells, it will contain chemical wood constituents, such as dissolved minerals, sugars, or other extractives, namely taxifolin, catechin, dihydrokaempferol and abietic acid [20–22]. These substances could have negative effects for aquatic ecosystems if the disposal of press water is not performed adequately [23, 24]. Among the PW of different wood species, e.g., Pine, Fir, Spruce, Poplar, and Beech, Douglas-fir showed the highest phenol index, chemical and biochemical oxygen demand [25]. Moreover the chemical oxygen demand (COD), which describes the amount of oxygen required to oxidize the organic material, of the tested PW was above 10,000 mg L^−1^ with an acidic pH, and none of the tested PWs showed a 100% degradability of the water-soluble organic substances (Zahn-Wellens-Test) with Douglas-fir PW having the least degradability with only 83% [25]. Therefore, similar to the effluents of pulp and paper mills, PW must be treated before being released into water bodies or even before entering the local wastewater treatment plants [26, 27]. Depending on the local regulations for wastewater disposal, the economic costs of PW treatment could offset the benefits associated with the reduced energy costs of mechanical drying compared to an exclusively thermal drying [18, 19]. Hence, alternative applications should be examined.

Instead of viewing it as a wastewater, the organically loaded PW could act as a low-cost substrate for enzyme production in fungal cultivations, thus becoming a by-product of the mechanical drying and not a waste. The ability to degrade lignocellulose is widespread in fungi of the Ascomycota and Basidiomycota phyla [28]. The ascomycete filamentous fungus *Trichoderma reesei* is a major producer of carbohydrate active enzymes (CAZymes), which are used for the conversion of plant biomass to sustainable fuels and chemicals [29–31]. The high cost of enzyme production as an important factor for the economic feasibility of biorefineries, especially for the production of biofuels, has driven the development of several hypercellulolytic mutant strains [32–34]. One of these strains, RUT-C30, has been extensively modified from its progenitor wild-type QM6a by several rounds of random mutagenesis and screening [35]. One critical change was found to be a truncation of the glucose repressor gene *cre1*, which was linked to the ability of the strain to produce cellulases and hemicellulases even in the presence of repressing carbon catabolites [36]. Additionally, wood-decaying basidiomycetes, which are typically classified as white rots or brown rots, have evolved alongside their plant hosts and developed distinct strategies for the degradation of lignin along cellulose and hemicelluloses [37–39]. White rots produce multiple lignin-degrading peroxidases, which allow them to degrade lignin, aside from other cell wall components like cellulose and hemicelluloses [40, 41]. On the other hand, brown rots leave lignin largely intact due to a lack of lignin-degrading peroxidases [40, 42]. These lignin-degrading enzymes make basidiomycetes interesting for industrial and biotechnological applications [43].

Generally, media for fungal enzyme production require high amounts of carbon and nitrogen sources. Aside from that, fungal media require considerable amounts of other macro nutrients, like sulfate, phosphate, K^+^, Ca^2+^ and Mg^2+^. Furthermore, several micro elements, like Fe^2+^, Mn^2+^, Zn^2+^, Co^2+^, are required. While necessary in much lower concentrations, these are nevertheless very important for the production of enzymes and other cofactors [44, 45].

The carbon source, which optimally serves both as an energy source and inducer molecule, accounts for a substantial portion of the cultivation costs. Therefore, the identification of abundant low cost residues, which display good rheological properties, are non-toxic, and induce cellulase production, have led to the utilization of various side products, like lactose and sugarcane bagasse [46, 47]. To this end, the PW could become an interesting side stream of the sawmill industry, which could have a huge potential to be used as media supplement in the production of cellulolytic enzymes.

For this study, we chose *Pseudotsuga menziesii* (Mirb.) franco (Douglas-fir) as the PW source. It displayed the highest organic load among other wood species [25]. Additionally, Douglas-fir is a promising tree species with favorable mechanical properties and appears to be well adapted to climate change [48, 49]. Douglas-fir might gain more importance in Central Europe in the coming years due to its small vulnerability to summer drought and volume growth per hectare that exceeds that of native European tree species [50–52].

**Fig. 1:**
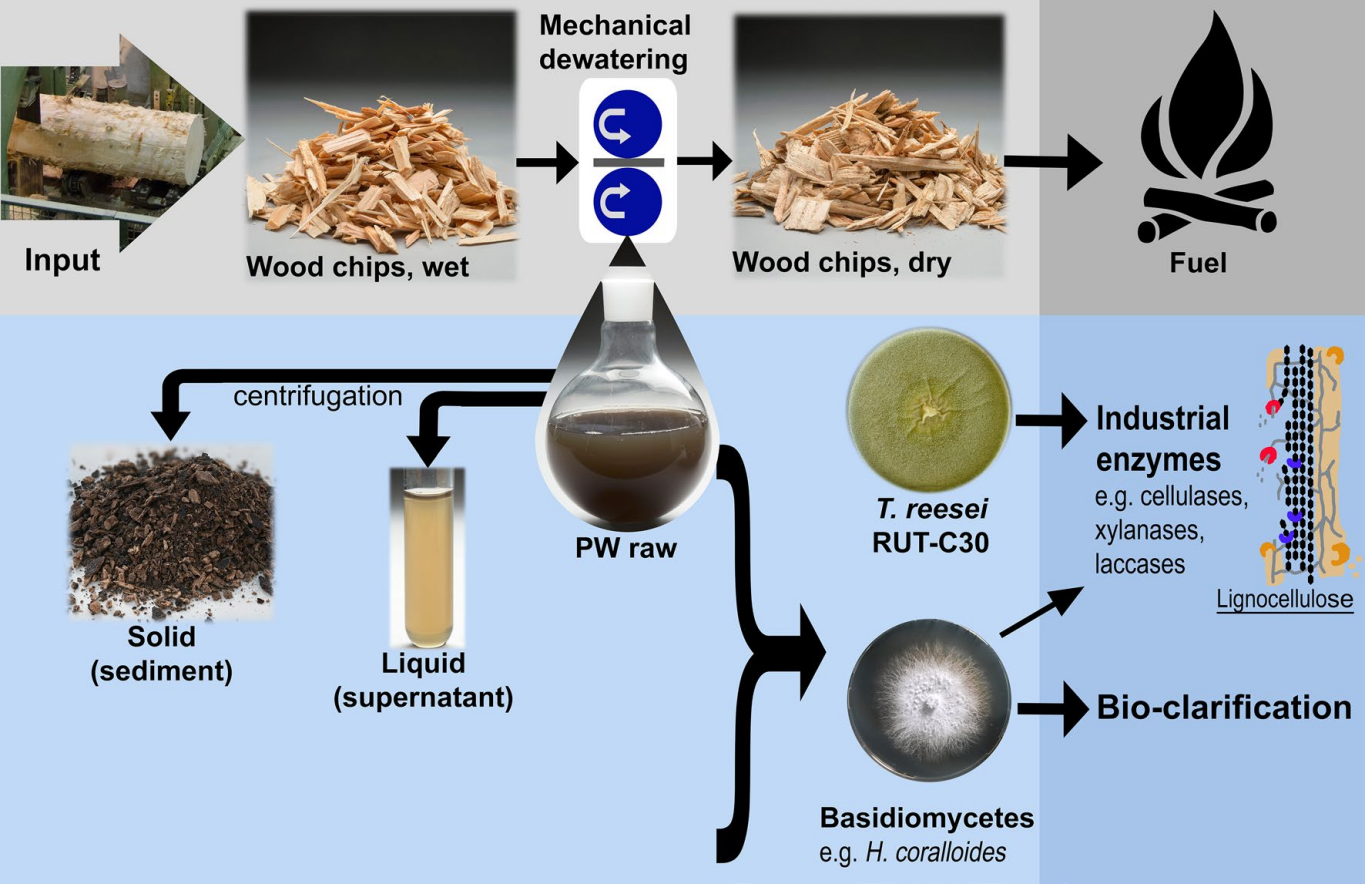
Schematic diagram of the processes and respective products associated with the mechanical drying of wood chips intended for energy production and the resulting side-product, press water (PW), tested here for its suitability in enzyme production with *T. reesei* RUT-C30 and bio-clarification with wood-degrading basidiomycete fungi

The aim of the present study was to analyze the potential of Douglas-fir-derived PW as a low-cost media supplement in the cultivation of wood degrading filamentous fungi. Since little is known about the PW composition, we initially investigated relevant physico-chemical characteristics of the PW, like dissolved ions, sugars, amount of solids and composition of the solids. We then tested the production of cellulases and xylanases with *T. reesei* RUT-C30 during growth on PW-supplemented media. Furthermore, the ability of several basidiomycetes to grow on PW-containing media and to clarify the aqueous medium containing suspended PW solids was investigated (Fig. 1).

## Results

### Chemical oxygen demand

The chemical oxygen demand (COD) of PW was analyzed to evaluate its organic load and the overall environmental impact. The results show that raw PW has a rather high organic load with a maximum of 10,750 mg L^−1^. The removal of macro solids from the PW by centrifugation lowered the COD to 8800 mg L^−1^. Ultimately, the high COD values of the PW are indicative for a high concentration of dissolved organic substances that are problematic in wastewater treatment but could be beneficial for fungal cultivations.

### Physico-chemical analysis

**Fig. 2:**
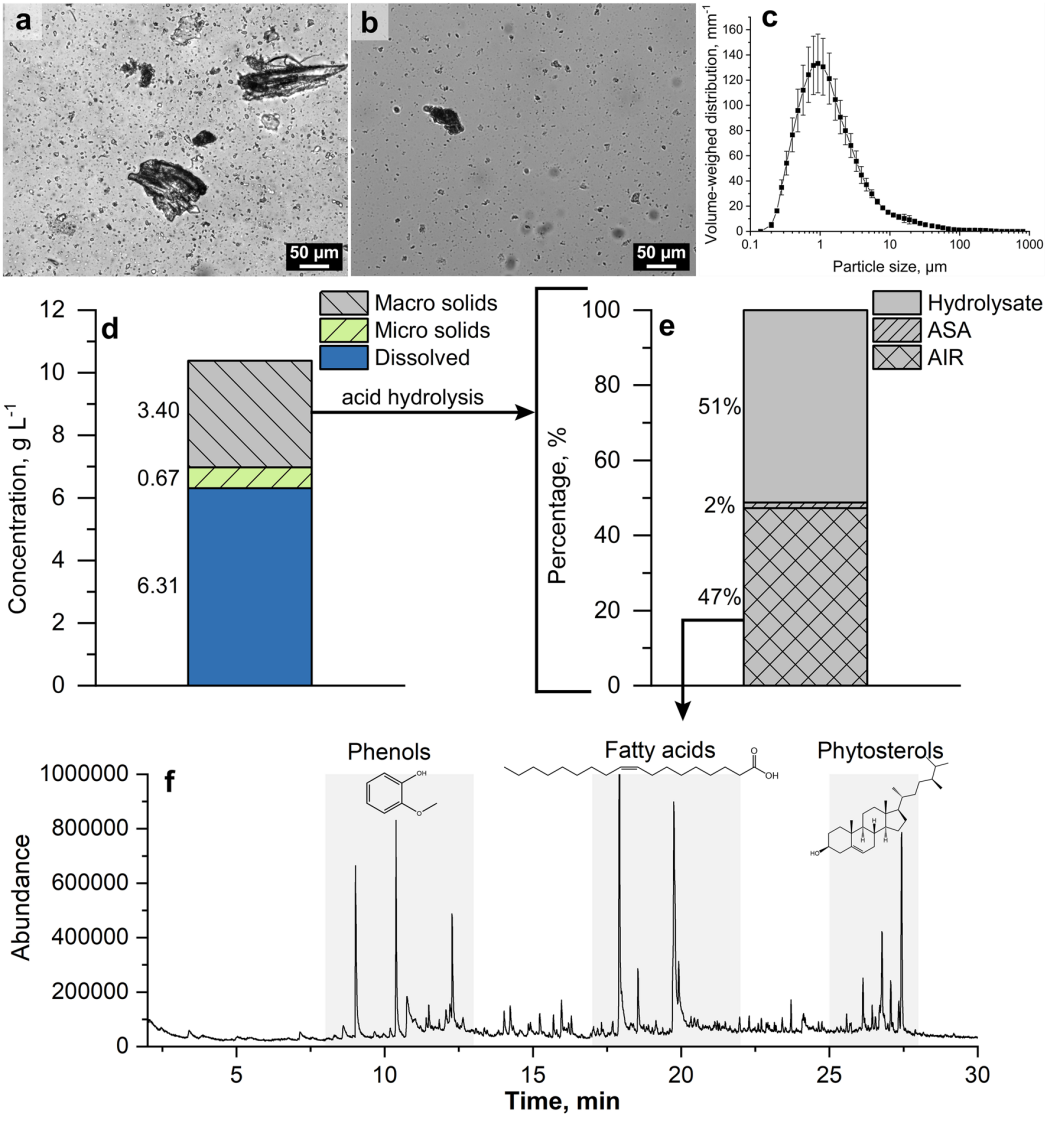
Microscopic photographs of raw PW (**a**) and PW supernatant (**b**). **c** Volume-weighed particle size distribution of Douglas-fir PW supernatant by laser-diffraction*.* **d** Concentration of macro solids, micro solids, and dissolved substances in the raw PW. **e** Mass percentage of the macro solids (from **d**) after acid hydrolysis, namely hydrolysate, acid soluble aromatics (ASA), and acid insoluble residues (AIR). **f** Chromatogram of the Py-GC/MS of AIR (from **e**) with the predominant compound classes with exemplary formulas for 2-methoxy-phenol, oleic acid, and campesterol

The analyzed raw PW was a heterogeneous suspension containing solids of different sizes and shapes (Fig. 2a). Even after centrifugation the liquid fraction remained turbid and colored, suggesting a high quantity of dissolved substances and suspended particles (Fig. 2b). Furthermore, the rather low pH of 4.42 indicates the presence of acidic organic substances.

The distribution of the volume-weighed particle size of the PW supernatant was measured by laser diffraction. The particles in the PW supernatant (after centrifugation at 4000 rcf for 15 min) showed a monomodal distribution, with most of the particles displaying a diameter of 1 µm and a maximum particle size of ca. 100 µm (Fig. 2c). Further separation of these suspended particles would require centrifugation at higher speeds, longer settling times or extensive filtration. The PW was fractionated by means of centrifugation and membrane filtration into sedimented macro solids, filtered micro solids, and dissolved substances, which were quantified gravimetrically. The total concentration of solids and dissolved substances in PW amounted to 10.38 ± 0.07 g L^−1^ (Fig. 2d). Dissolved substances represented the largest fraction of the PW with 6.31 ± 0.04 g L^−1^. The concentration of macro solids (at 3.40 ± 0.30 g L^−1^) and micro solids (at 0.67 ± 0.05 g L^−1^) together accounted for about 40% of the total dry mass.

An acid hydrolysis was performed to further investigate the composition of the macro solids in PW (Fig. 2e). Three fractions were obtained and quantified, namely hydrolysable polysaccharides (Hydrolysate), acid-insoluble residues (AIR), and acid-soluble aromatics (ASA). The hydrolysable polysaccharides in the solids of the PW accounted for 51.2% (w/w). Assuming that these are predominantly cellulose and hemicelluloses, a maximum of 1.73 g L^−1^ of the PW solids could potentially serve as a carbon source and inducer to produce cellulolytic enzymes in fungal fermentations.

To better elucidate the composition of the acid-insoluble residues [making up 47.3% (w/w)], we performed a pyrolysis GC/MS to identify the major pyrolysis products. The largest peaks were distributed in three main groups, corresponding to phenols (2-methoxy-4-methylphenol, 1,2-dihydroxybenzene, 2-methoxy-4-vinylphenol), fatty acids (oleic acid), and phytosterols (campesterol, stigmasta-3,5-diene) (Fig. 2f). The absence of levoglucosan was a good indication for the successful hydrolysis, since it is a pyrolysis product from carbohydrates. These results suggest that the polysaccharides in the PW sediment were effectively hydrolyzed leaving only the phenol-rich polymers and some resin derivatives behind.

### Dissolved phenolic compounds

To analyze the dissolved phenolic compounds in the PW we used a solid phase extraction cartridge (SPE). The methanol soluble fraction was analyzed using GC/MS. The chromatogram (Additional file 1: Fig. S5) showed the presence of some phenolic substances, like 3-(4-hydroxyphenyl)-1-propanol, and sugar derivatives. However, the flavonoid taxifolin was found to be the most abundant compound in the PW at a concentration of 7.4% (w/w) in the extract.

### Dissolved ions

The most abundant ions measured according to standard methods for the examination of water in the PW were potassium (K^+^; 130 mg L^−1^), calcium (Ca^2+^; 49.2 mg L^−1^), and SO_4_^2−^ (38 mg L^−1^) (Table 1), followed by iron (Fe^2+^; 20.10 mg L^−1^), magnesium (Mg^2+^; 14.60 mg L^−1^), manganese (Mn^2+^; 4.83 mg L^−1^), sodium (Na^+^; 4.28 mg L^−1^), and zinc (Zn^2+^; 0.61 mg L^−1^). As expectable from wood, PW was found to be a poor source of nitrogen with nitrate concentrations below detection range and only 1.43 mg L^−1^ of ammonium.

**Table 1:** Ion concentration in PW and relative to Mandels-Andreotti medium (MA)

	PW		MA	
	mg L^−1^	mM	mM	covered by PW, %
PO_4_^3−^	16.90	0.18	14.70	1.21
SO_4_^2−^	38.00	0.40	11.85	3.34
NH_4_^+^	1.43	0.08	21.19	0.37
NO_3_^−^	< 5	–	–	–
Na^+^	4.28	0.19	–	–
Ca^2+^	49.20	1.23	2.72	45.13
Mg^2+^	14.60	0.60	1.22	49.38
K^+^	130.00	3.33	14.70	22.62
Trace elements				
Fe^2+^	20.10	0.36	0.02	2001.60
Mn^2+^	4.83	0.09	0.01	874.88
Zn^2+^	0.61	0.01	0.005	184.86

### Sugar analysis

The most abundant monosaccharides in the PW measured using HPAEC-PAD were fructose and glucose with 0.60 g L^−1^ and 0.24 g L^−1^, respectively, followed by galactose (0.09 g L^−1^) and arabinose (0.03 g L^−1^). Xylose (13.81 mg L^−^1) and mannose (2.77 mg L^−1^) were the least abundant detectable sugars. The disaccharide concentrations of cellobiose and sucrose were 2.77 mg L^−1^ and 1.9 mg L^−1^, respectively. The combined concentration of mono- and di-saccharides in the PW amounted to 0.99 g L^−1^.

### Phenotype of *T. reesei* RUT-C30 on PW agar

To characterize the phenotype of *T. reesei* RUT-C30 on different media including or excluding raw PW, agar plates were inoculated with the same concentration of conidia and cultivated under identical conditions. Three types of Mandels-Andreotti (MA) salt-based solid media were prepared: plain MA agar without any carbon source, MA agar with 0.2 g L^−1^ glucose, and MA agar but without additional glucose. To observe the phenotype of *T. reesei* also on raw PW alone, one additional PW medium was prepared without MA salts as a control.

**Fig. 3:**
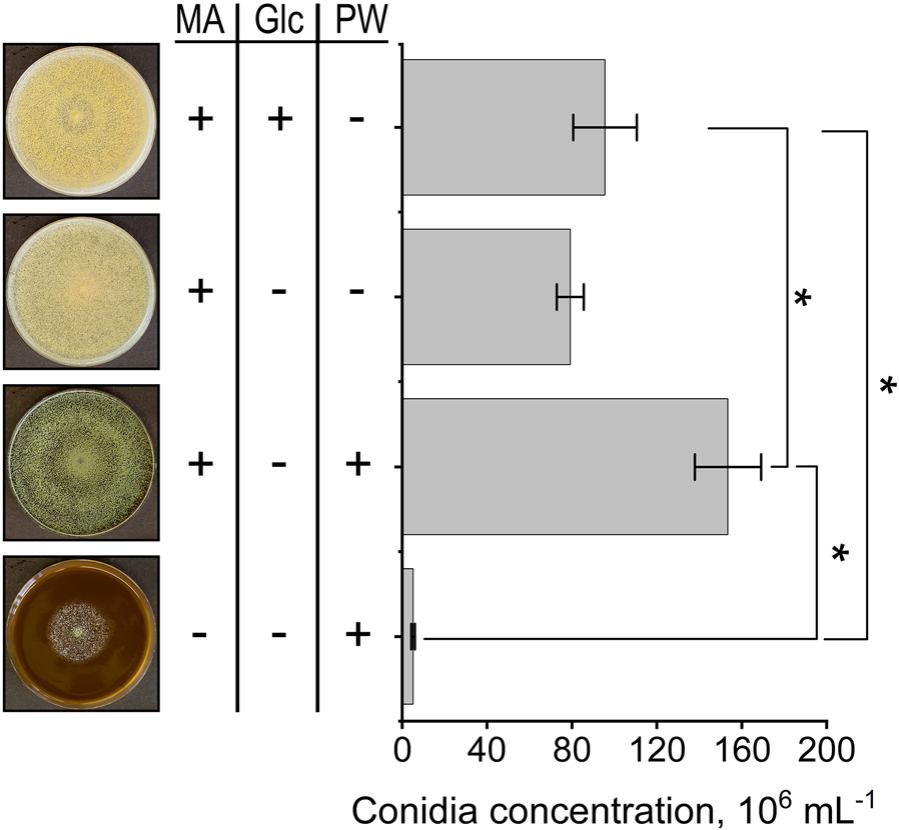
Spore concentration and phenotype of *T. reesei* RUT-C30 grown on various agars with combination of MA salts (MA), 0.2 g L^−1^ glucose (Glc), and raw press water (PW). Conidia were washed from the plates with equal amounts of water and quantified after 10 days, incubated at 25 °C in constant light

A clear phenotypic difference was observed, particularly regarding the color of the conidia, which turned green on PW (Fig. 3). Moreover, spore concentrations were much higher on the plates with PW. The addition of glucose to the MA agar only slightly increased sporulation, and PW medium without the addition of MA salts was not sufficient for optimal growth (Fig. 3).

### Liquid cultivation in PW supernatant with surplus of nutrients

In the following, we cultivated *T. reesei* RUT-C30 in liquid cultures with different concentrations of PW supernatant (after centrifugation) to observe the effects on endoglucanase and endoxylanase production. Here, the liquid cultures were formulated with the complete MA medium and with Avicel (microcrystalline cellulose) as a carbon source. The ion concentrations varied slightly, depending on the used PW supernatant volumes.

**Fig. 4:**
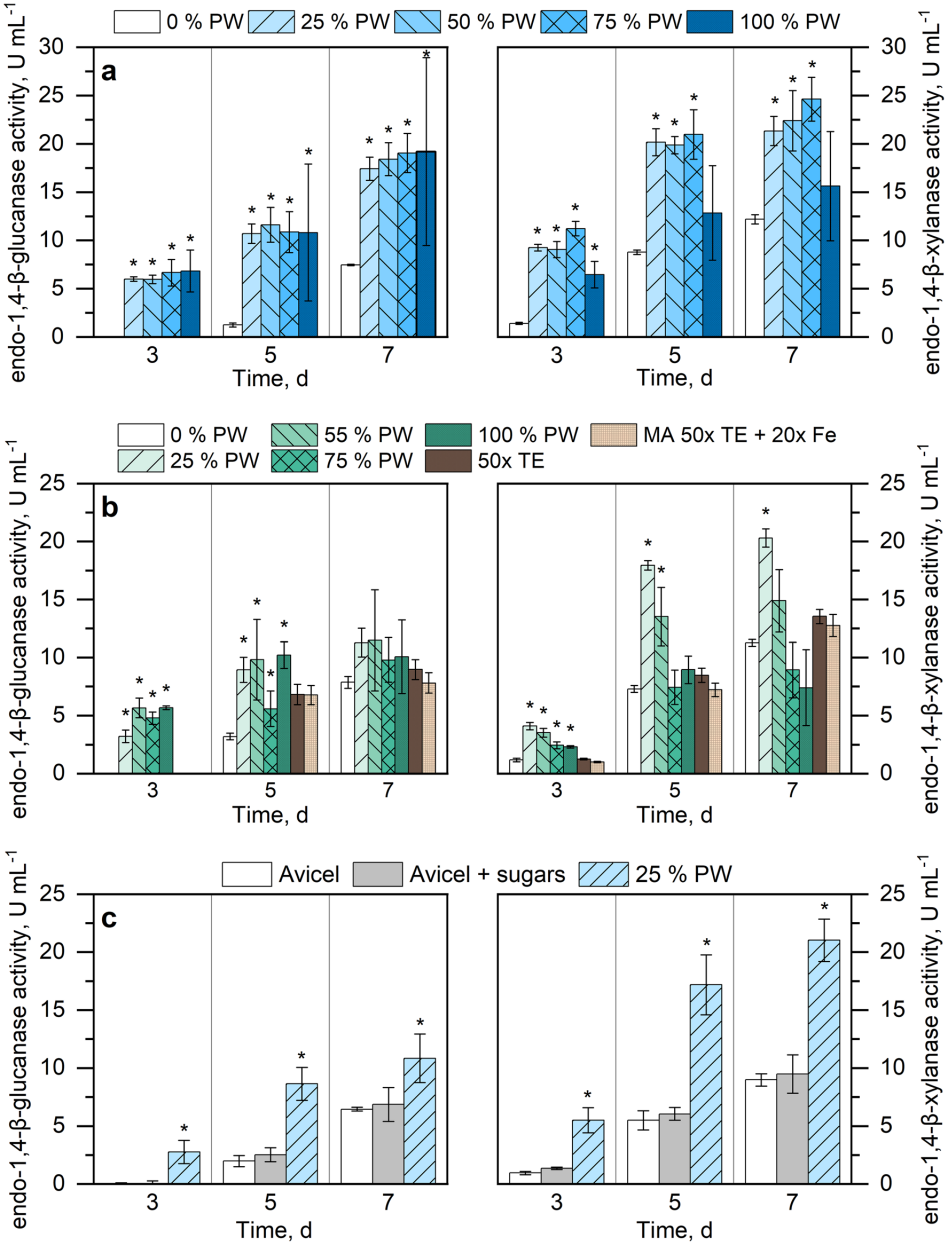
Endoglucanase and endoxylanase activities of *T. reesei* RUT-C30 cultivated in shaking flasks at 30 °C and 250 rpm. Avicel (microcrystalline cellulose) was used as a carbon source (1% w/v). **a** MA medium supplemented with different PW supernatant concentrations (25, 50, 75, 100%). **b** MA medium replacing salts (KH_2_PO_4_, MgSO_4_, CaCl_2_), trace elements, and Avicel according to the raw PW concentration. MA 50 × TE and 50 × TE + 20 × Fe are controls with increased trace element (TE) concentrations and iron, respectively. No trace elements were supplemented to raw PW concentrations starting at 55%. **c** MA medium with 1% (w/v) Avicel, 1% (w/v) Avicel + sugars based on the concentration in 25% PW, and 25% PW supernatant supplemented with MA medium. Error bars represent the standard deviation (n = 3). Significant differences (p > 0.05) relative to the control (0% PW) are indicated by asterisk

The enzymatic activities of the culture supernatants showed substantial enhancement in PW supernatant media compared to the control condition (Fig. 4a). Most noticeably, only the cultures supplemented with PW supernatant displayed substantial enzymatic activities already at day 3, whereas the control media with 0% PW needed several days more to reach the same level of activity. The activities seen in the culture supernatants with PW supernatant continued to be significantly higher than the controls throughout the cultivations, except for the endoxylanase activity in 100% PW supernatant, which was noticeably lower than the other PW conditions. However, the gap between the activities measured in 0% PW and 25–75% PW supernatant decreased over the course of the cultivation period. For instance, the endoglucanase activities measured in the PW supernatants were on average 8.8-fold higher at day 5 and only 2.5-fold higher at day 7 compared to the control without PW. Similarly, the endoxylanase activity measured between 25 and 75% PW supernatant displayed a 2.3-fold and 1.9-fold increase relative to the control at day 5 and 7, respectively. No significant difference in enzymatic activities was observed among the cultures with different PW supernatant dilutions. These results demonstrate that the addition of PW supernatant can robustly enhance the cellulase and xylanase activities in *T. reesei* RUT-C30 over a broad concentration window.

### Liquid cultivation in raw PW with replacement of salts and C-source

Since raw PW contains several nutrients in relevant quantities (mainly trace elements but also theoretically hydrolysable solids; Table 1, Fig. 2d), we wanted to test whether some of the components of the conventional fungal cultivation medium (MA) could be replaced by raw PW (with solids, not centrifuged). The minimal PW concentration at which the trace elements could be eliminated from the MA medium was 55% raw PW, as calculated based on the zinc concentration (Table 1). Since cobalt was not measured in the PW it had to be supplemented separately. However, at this dilution level, iron and manganese concentrations are higher than in the conventional MA medium. Particularly high concentrations of Fe^3+^ ions were reported to be inhibitory for the saccharification of cellulose in other fungi [53]. Therefore, to elucidate whether an overdose of trace elements would have negative effects, we prepared a control condition with 50 × trace elements. Furthermore, the carbon source Avicel was replaced based on the theoretically hydrolysable solids in the PW. The soluble monosaccharides were not considered in this case.

Comparable to the results seen on PW supernatant, the effect of the raw PW was most noticeable at day 3, where endoglucanase and endoxylanase activities of the cultures were significantly higher than the MA control (Fig. 4b). The highest endoglucanase activities were observed after 7 days of cultivation in 55% (11.5 ± 1.2 U mL^−1^) and 25% raw PW (11.3 ± 0.5 U mL^−1^). The endoxylanase activities showed a clear advantage at 25% PW, which yielded significantly higher activities than the other PW conditions, reaching a maximum activity of 20.3 U mL^−1^ after 7 days. Raw PW concentrations above 55% showed no significant enhancement of the endoxylanase activity.

The 50-fold increased concentration of trace elements in the medium led to overall slightly higher enzymatic activities, but the difference was only significant for the endoglucanase activities at day 5 compared to the 1 × control.

These results indicate that it is possible to replace some of the salts, Avicel, and trace elements present in MA medium by PW while maintaining the enhanced endoglucanase and endoxylanase activities up to 55% raw PW. However, as demonstrated by the 50 × trace elements control, the enhancement of enzymatic activity seen in the PW cultivations can only partially be explained by the increased concentration in salts or trace elements. Nevertheless, no significant inhibition was observed due to the surplus of iron.

### Liquid cultivation with addition of free sugars simulating 25% PW

To determine whether the free sugars present in the PW contribute to the observed enhanced enzymatic activities, we simulated the conditions present in the 25% PW supernatant by supplementing MA media with glucose, fructose, arabinose, galactose, and cellobiose in the respective concentrations. The addition of free sugars to the cultivation medium resulted in no significant difference compared to the control condition with only Avicel (Fig. 4c). On the other hand, the addition of 25% PW supernatant recurrently resulted in significantly higher enzymatic activities compared to the two conditions without PW. This suggests that the free sugars in PW are not responsible for the observed beneficial effects of PW during the cultivations.

### Liquid cultivation with PW solids as C-source

**Fig. 5:**
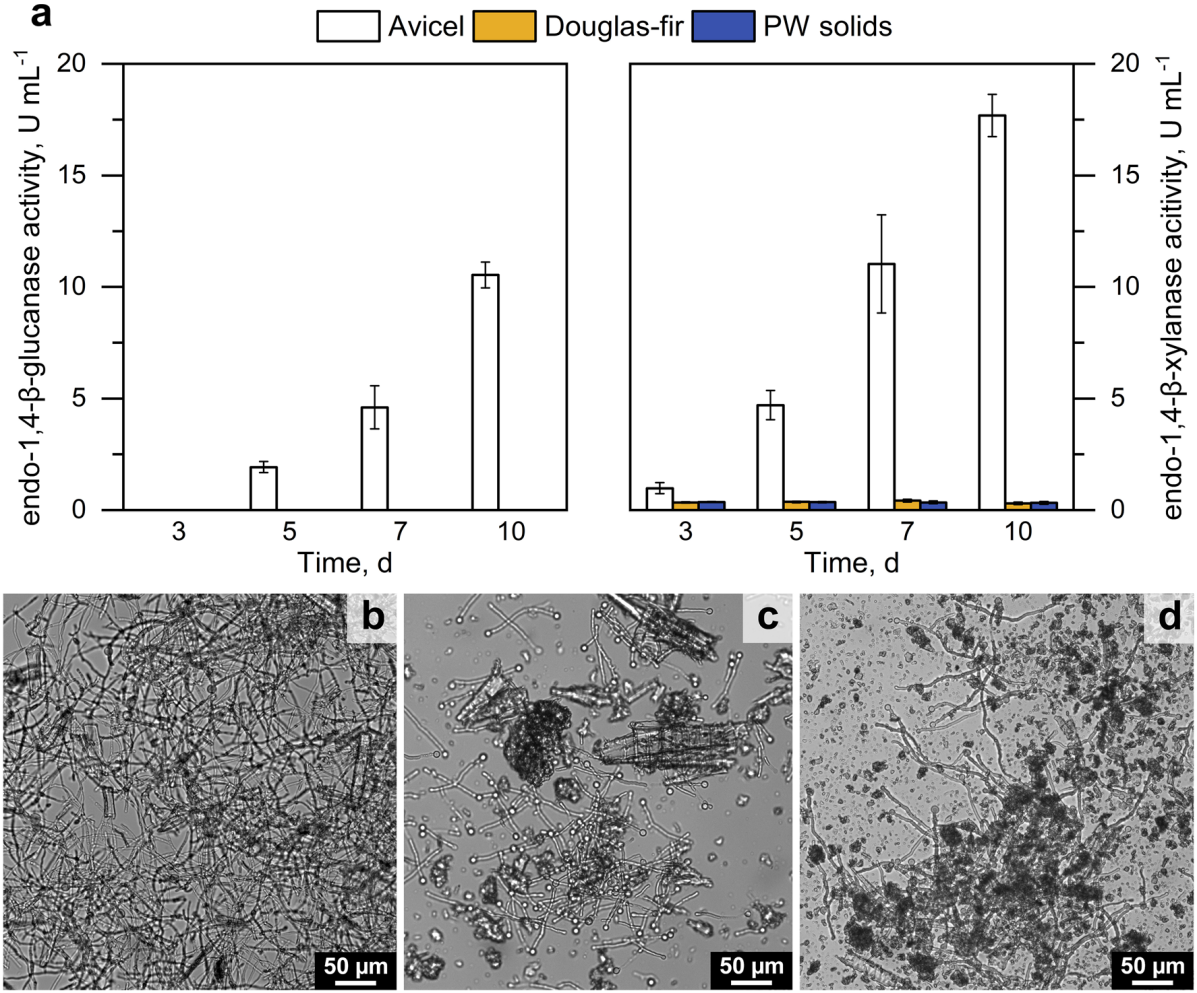
**a** Endoglucanase and endoxylanase activity of *T. reesei* RUT-C30 cultivated in MA medium with 1% (w/v) Avicel, Douglas-fir wood powder, and PW sediment. Shaking flasks cultivated at 30 °C at 250 rpm. Micrographs at 10 × of 5 day old cultures with **b** Avicel, **c** ball-milled Douglas-fir wood, **d** ball-milled PW solids (sediment). Error bars represent the standard deviation (n = 3)

To estimate to what extent *T. reesei* RUT-C30 can use the PW solids as a carbon source and inducer, liquid cultures were prepared with 1% ball-milled PW solids, non-pressed Douglas-fir wood and Avicel as standard carbon source. Although the fungus seemed to grow, there was a clear difference in the development of the fungal biomass in the liquid cultures with PW solids and Douglas-fir wood vs. the Avicel-grown cultures (Fig. 5b–d) and almost no enzymatic activities could be measured in the culture supernatants, even after 10 days (Fig. 5a). These results suggest that *T. reesei* is unable to utilize the PW solids or the Douglas-fir wood powder as a carbon source.

### Radial growth rate of several basidiomycetes in the presence of PW

**Fig. 6:**
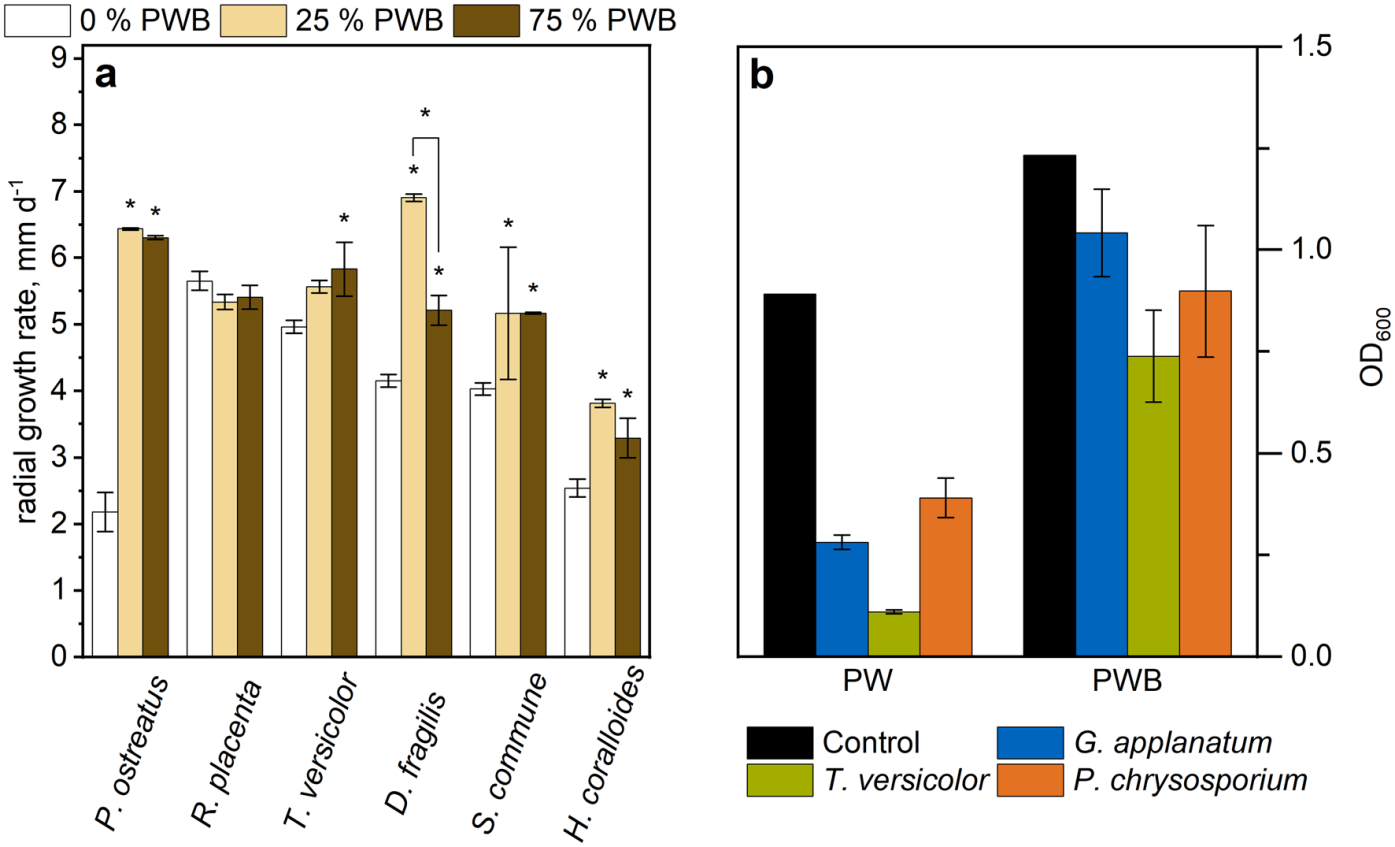
**a** Radial growth rate of several basidiomycetes on yeast malt extract agar supplemented with 25% and 75% (v/v) press water Douglas-fir with bark (PWB). Cultivation at 25 °C. Growth measured daily over a period of 2 weeks. Growth rate was calculated from the slope of the growth curve. Significance (p < 0.05) in relation to the control without PWB. **b** Turbidity (OD 600 nm) of culture supernatants incubated in shaking flasks 4 days at 28 °C and 100 rpm. Error bars represent the standard deviation of biological replicates (n = 3), thus the PW and PWB controls are displayed without error bars

The radial growth rate of a series of wood-degrading basidiomycetes was measured on agar plates containing yeast malt extract agar (YEMA) supplemented with PW from Douglas-fir (25 and 75% v/v) to assess the potential of wood PW as a substrate for lignocellulolytic fungi (Fig. 6a). Considering the ability of the tested strains to degrade lignin, we decided to use a batch of PW from Douglas-fir wood chips but with bark (PWB), which had a darker color and higher organic load (Additional file 1: Fig. S1).

We found that PWB enhanced the radial growth rates of all tested white rot fungi but not for the brown rot *Rhodonia placenta*. The strongest improvement was observed for *Pleurotus ostreatus*, for which the growth rate was accelerated from 0.18 mm d^−1^ (0% PW) to 0.54 mm d^−1^ (25% PWB), representing a three-fold improvement over YEMA. Despite showing a less drastic effect, a clear tendency of faster radial growth with increasing PWB concentrations was also observed for the used strains of *Dentipellis fragilis*, *Schizophyllum commune*, *Hericium coralloides* and *Trametes versicolor* (Fig. 6a).

### Bio-clarification of the PW

To assess the potential to reduce the turbidity of PW (with and without bark), as beneficial trait for biological wastewater treatment, we cultivated *T. versicolor*, *G. applanatum*, and *P. chrysosporium* in liquid cultures of PW supplemented with potato dextrose liquid medium (PDY) and measured the optical density of the culture supernatants. A turbidity reduction compared to controls incubated without fungal biomass was observed for all the tested strains in PW and PWB. The highest turbidity reduction was observed in *T. versicolor* cultures (Fig. 6b). Furthermore, all strains displayed a change in coloration of the PW, indicating the uptake, degradation or metabolization of some PW components (see Additional file 1: Figs. S3, S4).

### Discussion

The optimization of wood chip drying by mechanical dewatering in conventional sawmills could lead to higher energy efficiency and reduced fuel consumption [54]. However, the COD values of the resulting PW are comparable with effluents seen in the pulp and paper industry (thermo-mechanical pulp processing effluents and black liquor), which cause considerable operating costs due to the requirement of wastewater treatment [55–59]. Therefore, the treatment of large PW volumes could similarly become a financial burden. On the other hand, higher COD values, which translate to higher concentrations of substrates, would be beneficial for fungal fermentations. In parallel, PW utilization in the cultivations could reduce the need for freshwater, which is an important aspect for a more sustainable industry [60].

Wood type, felling season, and the location in which the trees have been growing ultimately define the composition and concentration of dissolved substances in the PW [61, 62]. Regarding soluble carbohydrates, glucose and fructose can be found as free sugars in the xylem of Douglas-fir, although it might vary depending on biotic and abiotic factors [62, 63]. The occurrence of cellobiose, the characteristic building block of cellulose, shows that there is a certain mechanical degradation of the cell wall, because it is not a naturally occurring disaccharide in wood [64]. Although Douglas-fir as a typical softwood contains more mannan than xylan (see Additional file 1: Table S2), we observed considerably lower mannose concentrations compared to xylose. Also the arabinose and galactose concentrations were higher than expected, indicating that these sugars might be more labile and sensitive to the mechanical stress [65–67].

One of the concerns of supplementing fungal media with PW was the possible presence of bioactive substances, like phenolic compounds, also lignin derivatives or tannins, that could inhibit fungal growth [68, 69]. The radial growth experiments on agar plates with the white rots *P. ostreatus*, *T. versicolor*, *D. fragilis*, *S. commune*, *H. coralloides* and the brown rot *R. placenta* were made using PWB as a supplement, which contained more dissolved substances than PW (Additional file 1: Fig. S1, Table S3). Altogether, no growth inhibition was observed on all the tested fungal strains, hence the PW of Douglas-fir was considered non-toxic for these fungi. Interestingly, while most white rots benefitted from the PW presence, the only brown rot strain that was tested, *R. placenta*, showed no significant growth rate change. While more species will have to be tested, it is intriguing to speculate that the differences seen among the basidiomycetes is related to the presence or absence of ligninolytic enzymes [39, 40]. A deeper examination of the effect of PWB components on the transcriptome of white rots would be required to determine the effect of PW on the expression of lignin-degrading peroxidases of basidiomycetes.

In contrast to the wood-degrading basidiomycetes, *T. reesei* RUT-C30 lacks the capacity to degrade lignin [70]. Considering also that bark is the most extractive-rich tissue of Douglas-fir, the cultivations of *T. reesei* were made using PW from debarked wood chips, thus minimizing any unforeseen interaction with potential inhibitors [21, 71, 72]. At low PW concentrations (25–55% PW) the enhancing effect for enzyme activity was similar for the cultivation with raw PW and PW supernatant. The enzymatic activity of the cultures with PW was consistently higher after 3 days of cultivation compared to the control, even after the replacement of salts and trace elements (Fig. 4b), suggesting that the addition of 25–55% PW to the cultivation medium has a stronger effect than varying the ion concentrations (Fig. 4a, b). On the other hand, at higher concentrations (75–100% PW) the measured enzymatic activities were less consistent, the endoxylanase activity being most affected by the increased concentration of raw PW (Fig. 4b) compared to PW supernatant (Fig. 4a). Since the ion concentrations had no measurable effect on the enzymatic activities, the only difference between the PW supernatant and raw PW was the presence of PW solids (sediment).

The compounds found in the acid-insoluble residues of PW sediments during the Py-GC/MS correspond to typical lignin fragments found in Douglas-fir wood (Fig. 2f) [73, 74]. Lignin has been suggested to bind hydrophobic faces such as on the cellulose-binding module of cellulases, thus inactivating the enzymes by non-productive binding [75–78]. Consequently, it is possible that enzymes could bind to the surface of the PW sediment, thus reducing the enzymatic activity of the culture supernatants at higher concentrations of raw PW (Fig. 4) [77]. Some small phenolics and one characteristic flavonoid, taxifolin, were found in the PW (see Additional file 1: Fig. S5). Several phenolic compounds generated from lignin degradation have been reported to inhibit endoxylanases in a non-competitive multisite mechanism [79]. However, contrary to reports, no substantial inhibition of endoglucanase activity and only a slight reduction of endoxylanase activity was observed for *T. reesei* RUT-C30 (see Additional file 1: Fig. S2) [71, 80–82]. Interestingly, phenolic substances in the black liquor from the Kraft pulping process have been reported to modify the protein structure of commercial xylanases, enhancing the hydrolysis of xylan [83]. This might have contributed to the enhanced activities seen in liquid cultures at low PW concentrations (25–55%), while at higher PW concentrations (75–100%) the positive effects are offset by the non-productive binding on the PW sediment and inhibition by other substances. Nevertheless, the plate-based assays showed that the concentration of potentially inhibiting compounds even in 100% PW are insufficient to cause a complete inhibition of *T. reesei* growth (Fig. 3).

The lack of nutrients, especially a nitrogen source, had a stronger influence on the growth of *T. reesei* RUT-C30 than the presence of PW, as observed on agar plates (Fig. 3). However, this is a common issue among lignocellulosic substrates from agro-industrial wastes, such as sugarcane bagasse, soybean hulls, pulp and paper sludges, and even pretreated wood chips [32, 84–86], the only exception being manure [87]. While PW is also not a rich source of cellulose and hemicelluloses compared to the aforementioned substrates, several waste streams have their own limitations regarding the production of cellulolytic enzymes. In the case of waste paper sludge, for example, nonproductive binding of the substrate or inhibition from mineral paper additives often render this substrate unsuitable for enzyme production [84–88]. Similarly, steam-pretreated wood can contain inhibiting sugar degradation products like furfural and hydroxymethylfurfural [86].

The carbon source and inducer molecules constitute a major cost factor in the production of lignocellulolytic enzymes, and thus finding cheaper alternatives would be beneficial [32, 89]. Sophorose and lactose are favored supplements for the production of cellulases in *T. reesei* as soluble inducers and carbon sources [90]. However, sophorose is prohibitively expensive and lactose, although cheap and largely available, induces only a limited amount of cellulases compared to cellulosic substrates [91–94]. The (partial) replacement of Avicel by the solids in PW would have had the potential to reduce some of the costs. However, at least for *T. reesei* RUT-C30, this was found to be impracticable, since it was unable to grow on PW sediment as sole carbon source (similarly to non-pressed Douglas-fir wood powder; Fig. 5a), suggesting that the mechanical treatment of the wood particles in the PW do not open these up sufficiently [70]. This observation is in line with reports about *Trichoderma *spp. preferably growing on wood that has been previously degraded or pre-treated either chemically or by other fungi [95, 96].

Cellobiose and further cellulose-derived oligosaccharides are known to induce the production of cellulases in *T. reesei* and other filamentous fungi [97–99]. However, neither the dissolved sugars nor the suspended solids in the PW were found to be causative for the positive effects, and the minerals had only a small positive influence (Fig. 4). Therefore, we suggest that the enhancing effect might come from other, so far unidentified, organic molecules dissolved in the PW. There is a possibility that some components in the PW also directly interact with some signaling pathways or induce some modifications on the hyphal cell walls, similar to N,N-dimethylformamide, which was reported to enhance cellulase production via calcium signaling and permeabilization of the hyphal cell wall in *T. reesei* RUT-C30 [100]. It has also been suggested that the micromorphology of *T. reesei* influences the enzyme productivity [101]. While this was not part of this study, an analysis could reveal if the PW affects the hyphal structure (Fig. 4b). A deeper understanding of the composition of the organic fraction of the PW combined with an RNAseq analysis of *T. reesei* RUT-C30 in presence of PW would furthermore allow to identify internal signaling processes to elucidate how the PW interacts with the fungus.

One interesting alternative to the conventional wastewater treatment that might offer a quick solution for the high turbidity and high COD would be fungal-assisted bio-clarification of wood PW [102]. Our results verified that basidiomycete fungi can substantially reduce the turbidity after a few days of cultivation (Fig. 6b). In addition, we observed a decrease in absorption over the entire visible spectrum (230–800 nm) (Additional file 1: Fig. S3), indicating parallel degradation of many dissolved substances probably by secreted ligninolytic enzymes, like laccases [103–105], which represents an additional benefit but nevertheless needs to be verified.

## Conclusions

The PW of Douglas-fir wood chips is a complex sawmill side stream with a high COD, demanding costly wastewater treatment. However, several of the dissolved components as well as the solids are also part of conventional fungal growth media, and despite the presence of potential phenolic inhibitors, the PW was found to be a suitable non-toxic media supplement for several basidiomycetes as well as the industrially employed ascomycete *T. reesei*. The supplementation of cleared PW to liquid cultures of *T. reesei* RUT-C30 reduced the lag-phase and significantly enhanced the endoglucanase and endoxylanase activities in the supernatant. The supplementation of 55% raw PW to the cultivation media allowed the replacement of 100% trace elements (Fe^2+^, Mn^2+^, Zn^2+^) of the conventional MA medium as well as 12, 13 and 6% of Ca^2+^, Mg^2+^, and K^+^, respectively, without losing the positive influence on enzyme production. Furthermore, PW allowed to replace an equal volume of fresh water. The utilization of raw PW in fungal cultivations could therefore combine a bio-clarification of this sawmill effluent with the creation of added-value by lowering costs of media formulations and an increased product yield.

A further analysis of the molecular pathways being activated by PW as well as an advanced qualitative and quantitative chemical analysis will be crucial to understand the mechanisms of the positive effects seen in this study and pinpoint the responsible compounds.

## Material and methods

### Press water samples

The press water used in this study was obtained by pressing debarked wood chips of *Pseudotsuga menziesii* (Douglas-fir) with a roller press (wood chips squeezer) located at Bohnert Technik GmbH in Seebach, Germany. Douglas-fir wood chips with and without bark were obtained from local sawmills. The wood chips were fed evenly on a patented plate conveyor chain, which passes between two large rollers [106]. The chain helps deliver the wood chips to the pressing zone and at the same time facilitates the drainage of press water through narrow gaps between the chain links. The press water (PW) was collected directly beneath the pressing zone in large container. The PW was thoroughly homogenized before fractionation into 1 L HDPE bottles. The press water was always stored at − 20 °C if not otherwise mentioned.

### Physico-chemical analysis of PW

The raw PW was centrifuged at 4000 rcf for 15 min in a centrifuge (Heraeus Megafuge 40R, Thermo Scientific). The PW supernatant was carefully decanted, aliquots of 100 mL were transferred to lyophilisation flaks, and the same was done for the sediment. The total mass content was determined gravimetrically after drying the samples in a freeze dryer (Christ Alpha 1-2LDplus). Fine particles that remained suspended after the centrifugation were quantified gravimetrically using cellulose acetate filter membranes with pore size of 0.45 µm (Sartorius).

### Particle size distribution

A laser diffraction system HELOS (Sympatec, Germany) with the RHODOS dispersing unit was used to measure the particle size distribution over a wide range of sizes of the PW supernatant.

### Chemical oxygen demand

The chemical oxygen demand (COD) and dissolved ions concentrations were measured by the Chair of Urban Water Systems Engineering at the Technical University of Munich, according to the German standard methods for the examination of water [107–110].

### Acid hydrolysis

The determination of acid insoluble residues in the sediments of the press water was conducted according to the standard procedures TAPPI T 249 and T 222. Briefly, 1 g of the lyophilized and homogenized PW sediments was incubated for 2 h with 15 mL of 72% sulphuric acid in a water bath at 20 °C. Then, the acid was diluted to 3% and incubated for 4 h at 100 °C. After hydrolysis the sample was filtered, the acid-insoluble residue (AIR) was determined gravimetrically and the acid-soluble aromatics (ASA was determined spectroscopically at 205 nm (ε 110 g L^−1^ cm^−1^) [111–113].

### Py-GC/MS and GC/MS analysis and sample preparation

Solid samples were measured using the pyrolysis gas chromatography coupled to a mass spectrometer (Py-GC/MS), VLMSD 5975C (Agilent Technologies) equipped with a VF17 MS 30 m × 250 µm × 0.25 µm column (Agilent Technologies). The pyrolysis was performed in a single shot analysis at 450 °C for 0.2 min. For liquid samples, PW was filtered followed by an extraction step using a SPE C18ec cartridge and methanol to elute the hydrophobic substances (Chromabond, Macherey–Nagel). The GC/MS conditions: Inj. 300 °C with a split of 40:1 and temperature program: T_1_ = 100 °C for 1 min, R = 10 °C/min, T_2_ = 300 °C for 4 min. Liquid samples were silylated prior to injection. The mass spectra were evaluated using the NIST MS library (NIST20). Taxifolin, was verified and quantified using a calibration curve with a reference (Sigma-Aldrich).

### HPAEC-PAD analysis of the PW

Free neutral sugars (list all sugars) were determined on a Dionex ICSW 3000 HPAEC-PAD instrument setup with a Dionex AS Autosampler, a Dionex gradient mixer GM-3 (Dionex Corp., California USA) and a CarboPacPA1 preparative IC column (4 × 250 mm) equipped with a CarboPacPA1 standard bore guard column (4 × 50 mm) (Thermo Fisher Scientific Inc., Massachusetts USA). The analysis was carried out with a 27 min isocratic method with a 10 mM sodium hydroxide solution for monosaccharides in deionized water with low total organic carbon at 1 mL min^−1^ flow rate and 30 °C. For the analysis of disaccharides, the sodium hydroxide concentration was elevated to 100 mM.

The PW samples were filtered with a PES membrane with a pore size of 0.2 µm and cleaned using an anion exchange SPE cartridge (Strata XA, Phenomenex) following the protocol of the manufacturer. The samples were further diluted with ddH_2_O before measurement in duplicate.

### Strain cultivation

*T. reesei* strain RUT-C30 was propagated on potato dextrose agar (Carl Roth) plates in the dark at 30 °C for two days, then switched to constant light at 25 °C for conidiation. All liquid media cultivations were carried out in 250 mL flasks without baffles containing 50 mL medium and shaken at 250 min^−1^ (25 mm throw) and at 30 °C in darkness, if not otherwise mentioned. For the inoculation, a respective volume of conidial suspension was added after optical density measurement to a final concentration of 10^6^ conidia mL^−1^. Cultures were always grown in triplicates if not otherwise mentioned.

*Schizophyllum commune* and *Ganoderma applanatum* were isolated by Philipp Benz by the Isar river between Moosburg and Freising, Germany. *Dentipellis fragilis* and *Hericium coralloides* were isolated by Markus Blaschke in the natural forest reserve Gitschger, Germany. The isolated strains were identified by sequencing the internal transcribed spacer (ITS) [114]. The *Pleurotus ostreatus* FPRL 40C, *Trametes versicolor* BAM116 (CTB863) and *Rhodonia placenta* BAM 113 (FPRL 280) were obtained from the German Federal Institute for Materials Research (BAM). *Phanerochaete chrysosporium* (DMSZ 1556) was obtained from the German Collection of Microorganisms and cell Cultures GmbH. Basidiomycete strains were cultivated on yeast malt extract agar (YMEA) containing 10 g L^−1^ glucose, 5 g L^−1^ peptone, 3 g L^−1^ malt extract, 3 g L^−1^ yeast extract and 20 g L^−1^ agar until the mycelium covered the entire plate.

### Phenotype analysis and growth rate on PW agar

The agar plates used for the phenotype experiments consisted of 2% agar adjusted to pH 5.0. The PW agar was prepared either without or with the addition of Mandels-Andreotti (MA) medium components and no addition of glucose [44]. MA minimal media agar was prepared with 0.2 g L^−1^ glucose to simulate the glucose concentrations present in the PW. The plates were inoculated with 2 µL from a spore solution containing 0.5 × 10^6^ conidia mL^−1^.

After 10 days of cultivation at 25 °C with constant light. The conidia were harvested by washing the agar plates with 5 mL H_2_O ten times covering one half of the plate and then repeating the procedure with another 5 mL of H_2_O for the other half of the plate. Conidia were filtered with glass wool, centrifuged, and finally resuspended in 5 mL H_2_O. The quantification was done by measuring the OD at 600 nm.

For the growth analysis of the Basidiomycete strains, 5 mm plugs were cut out of pre-cultures using a coring tool and then transferred to the middle of new plates with YMEA with no PW, with 25% or 75% (v/v) PW from Douglas-fir wood chips with bark (Additional file 1: Figure S1). All plates were prepared with 20 mL agar and the plugs were cut from the peripheral growth zone of the fungal cultures. The plates were incubated at 25 °C. The growth was observed daily and recorded with digital photographs using a camera equipped with a 60 mm macro lens (Nikon) next to a reference scale. The growth rate was calculated from the fitted curve of biological triplicates and technical replicates were measured in different directions.

### Press water liquid media for cellulase production

For the cultivation with PW supernatant, the raw PW was centrifuged at 4000 rcf for 15 min (Heraeus Megafuge 40R, Thermo Scientific) and the supernatant was decanted to be used in the liquid medium. The PW supernatant was diluted with ddH_2_O to different concentrations, namely 25, 50, 75, and 100% (v/v). The complete MA medium was added to each PW condition, so that only ddH_2_O was replaced in each condition. All liquid media were adjusted to pH 5.0 with 0.1 M phosphate-citrate buffer and 1% (w/v) Avicel PH-101 (Sigma-Aldrich) was used as a carbon source and inducer, if not otherwise mentioned.

The sedimented PW solids, after the centrifugation step mentioned above, were used as a carbon source, and compared to Avicel and wood powder obtained from unpressed Douglas-fir wood chips. The PW sediment was washed with ddH_2_O and dried overnight in a drying oven. To achieve a particle size like that of Avicel (500 µm), we treated the PW sediment and the Douglas-fir powder in a ball-mill (MM200, Retsch). Each flask with MA medium contained 1% (w/v) of the respective solid as a carbon source.

The cultivation with PW with replacement of salts and carbon source was carried out with uncentrifuged PW in different concentrations. The percentage of salts, trace elements and Avicel to be replaced were calculated based on the molar concentrations of dissolved ions (Table 1) and the total hydrolysable sediment in the PW (Fig. 2e). A PW concentration of 55% (v/v) was chosen as the minimal concentration needed to achieve a complete replacement of trace elements. No trace elements were added to the conditions containing more than 55% PW. Only (NH_4_)_2_SO_4_, peptone, urea, and CoCl_2_ were not replaced in any condition (Table 2).

**Table 2:** Mandels-Andreotti medium constituents modified for the cultivation of *T. reesei* with raw PW (% v/v) with replacement of Avicel

Components	Unit	MA media		raw PW media			
		1x	50 × TE	25%	55%	75%	100%
Salt solution							
KH_2_PO_4_	g L^−1^	2.00	2.00	1.89	1.75	1.66	1.55
CaCl_2_·2H_2_O	g L^−1^	0.40	0.40	0.35	0.3	0.26	0.22
MgSO_4_·7H_2_O	g L^−1^	0.30	0.30	0.26	0.22	0.19	0.15
Trace elements							
FeSO_4_·7H_2_O	mg L^−1^	5.00	250 (350^a^)	5.00	–	–	–
MnSO_4_·H_2_O	mg L^−1^	1.60	80	1.60	–	–	–
ZnSO_4_·7H_2_O	mg L^−1^	1.40	70	1.40	–	–	–
Carbon source							
PW	(v/v) %	–		25	55	75	100
Avicel	g L^−1^	10		9.57	9.05	8.7	8.27

Cobalt had to be supplemented to the medium, since considerable differences in the cellulase. 50 × TE: 50 times the concentration of trace elements (TE) was used as a control

^a^Iron concentration used in the iron-enriched control medium MA 50 × TE + 20 × Fe

The addition of free sugars to the cultivation medium was based on the sugar concentration measured in the PW. Glucose (0.061 g L^−1^), fructose (0.150 g L^−1^), galactose (0.022 g L^−1^), arabinose (0.007 g L^−1^), cellobiose (0.003 g L^−1^), xylose (0.003 g L^−1^), concentrations that correspond to 25% PW, were added to MA medium with 1% Avicel and autoclaved. MA medium with no sugars and 25% PW supernatant, both with 1% Avicel, were used as control.

### Enzymatic assays

Endo-1,4-β-d-glucanase and endo-1,4-β-d-xylanase activity assays were carried out according to the protocols (S-ACMC and S-AXBL) of the manufacturer (Megazyme, Ireland), slightly modified and adapted for a 96-well microplate. Each sample was tested in technical duplicate. The mean and the standard deviation are calculated from the biological triplicates and technical duplicates.

Inhibition of the enzymatic reactions in the presence of PW was verified using the same method as described above. As enzyme we used supernatant of a 5-day old *T. reesei* RUT-C30 culture grown with 1% Avicel. Each reaction consisted of 10 µL enzyme and increasing concentrations of PW, namely 20% (v/v) and 60% (v/v). The reaction volume was adjusted to 25 µL with ddH_2_O. These tests were performed in 6 technical replicates for each condition.

### Bio-clarification of PW

Liquid pre-cultures of *T. versicolor*, *G. applanatum* and *P. chrysosporium*, were made in shaking flasks with 50 mL potato dextrose yeast (PDY) in 250 mL Erlenmeyer flasks until enough biomass was formed. These strains were chosen due to their fast growth in liquid media. The biomass was then homogenized using an Ultra Turrax (IKA Werke GmbH, Germany) and transferred to the PDY media containing 50% PW or PWB. The cultures were inoculated with 10% biomass suspension and were cultivated in 100 mL Erlenmeyer flasks with 30 mL medium for 4 days at 28 °C, 100 rpm (50 mm throw). As a control condition PW and PWB flasks without fungal biomass were incubated together with the cultures. The entire volume of the cultures was collected in centrifugation tubes and centrifuged 2 min at 1000 rcf. The supernatants were carefully transferred to cuvettes and the OD was measured at 600 nm to calculate the turbidity change against the control condition.

### Statistical analyses

Statistical analyses were performed by applying one-way repeated measures analysis of variance (ANOVA) followed by Dunnett’s test using the growth condition without PW as control in OriginPro 2021 (OriginLab Corporation). Significance level of p < 0.05.

## Supplementary Information

**Additional file 1: Figure S1.** Physico-chemical analysis of press water from Douglas-fir with bark (PWB). **a** Concentration of macro solids, micro solids and dissolved substances. **b** Mass percentage of hydrolysate, acid soluble aromatics, and acid insoluble residues of PWB solids after acid hydrolysis. **Figure S2.** Endoglucanase and endoxylanase activities of *T. reesei* RUT-C30 culture supernatants in the presence of increasing PW (% v/v) concentrations. Cultivation in MA medium with 1% Avicel at 30 °C and 250 rpm. Significant differences (p > 0.05) relative to the control are indicated by asterisks. **Figure S3.** Absorbance scan of the culture supernatants of *G. applanatum*, *T. versicolor*, and *P. chrysosporium* cultivated in 50% PW with potato dextrose yeast medium (PDY) in shaking flasks with 30 mL medium, at 100 rpm (50 mm throw), 28 °C. Absorbance in the range of 230–800 nm was measured in UV transparent cuvettes. Error bars indicate the standard deviation of biological triplicates. The control was not measured in replicates. **Figure S4.** Photograph of the culture supernatants of *G. applanatum*, *T. versicolor*, and *P. chrysosporium* cultivated in 50% PW with potato dextrose yeast medium (PDY) in shaking flasks with 30 mL medium, at 100 rpm (50 mm throw), 28 °C. **Figure S5.** Chromatogram of the GC/MS of the hydrophobic fraction of dissolved substances in the PW, concentrated with a C18ec SPE cartridge. Chemical formulas for 3-(4-Hydroxyphenyl)-1-propanol and taxifolin are showed. N.i., not identified. **Table S1.** Ion concentration in PW Douglas-fir with bark relative to Mandels-Andreotti medium (MA). **Table S2.** Compositional analysis of Douglas-fir wood chips (pressed and unpressed) expressed in % of the total weight. **Table S3.** Concentration of dissolved sugars in Douglas-fir PW with bark measured in a HPAEC-PAD.
