# Supplementary material for: Press water from the mechanical drying of Douglas-fir wood chips has multiple beneficial effects on lignocellulolytic fungi
Source: Fungal Biol Biotechnol. 2022 May 23;9:10. doi: 10.1186/s40694-022-00141-y (PMC9128199; doi:10.1186/s40694-022-00141-y)
Supplement: Supplementary file 1 — Additional file 1: Figure S1. Physico-chemical analysis of press water from Douglas-fir with bark (PWB). a Concentration of macro solids, micro solids and dissolved substances. b Mass percentage of hydrolysate, acid soluble aromatics, and acid insoluble residues of PWB solids after acid hydrolysis. Figure S2. Endoglucanase and endoxylanase activities of T. reesei RUT-C30 culture supernatants in the presence of increasing PW (% v/v) concentrations. Cultivation in MA medium with 1% Avicel at 30 °C and 250 rpm. Significant differences (p > 0.05) relative to the control are indicated by asterisks. Figure S3. Absorbance scan of the culture supernatants of G. applanatum, T. versicolor, and P. chrysosporium cultivated in 50% PW with potato dextrose yeast medium (PDY) in shaking flasks with 30 mL medium, at 100 rpm (50 mm throw), 28 °C. Absorbance in the range of 230–800 nm was measured in UV transparent cuvettes. Error bars indicate the standard deviation of biological triplicates. The control was not measured in replicates. Figure S4. Photograph of the culture supernatants of G. applanatum, T. versicolor, and P. chrysosporium cultivated in 50% PW with potato dextrose yeast medium (PDY) in shaking flasks with 30 mL medium, at 100 rpm (50 mm throw), 28 °C. Figure S5. Chromatogram of the GC/MS of the hydrophobic fraction of dissolved substances in the PW, concentrated with a C18ec SPE cartridge. Chemical formulas for 3-(4-Hydroxyphenyl)-1-propanol and taxifolin are showed. N.i., not identified. Table S1. Ion concentration in PW Douglas-fir with bark relative to Mandels-Andreotti medium (MA). Table S2. Compositional analysis of Douglas-fir wood chips (pressed and unpressed) expressed in % of the total weight. Table S3. Concentration of dissolved sugars in Douglas-fir PW with bark measured in a HPAEC-PAD. [file 40694_2022_141_MOESM1_ESM.docx]

**Additional file**

**Growth-promoting effects of press water from the mechanical drying of Douglas fir wood chips on lignocellulolytic fungi**

Manfred J. Reppke^1†^, Rebecca Gerstner^1^, Elisabeth Windeisen-Holzhauser^2^, Klaus Richter^2^, J. Philipp Benz^1,3*^

*Correspondence: [benz@hfm.tum.de](mailto:benz@hfm.tum.de)

^1^Professorship of Fungal Biotechnology in Wood Science, Holzforschung München, TUM School of Life Sciences, Technical University of Munich, Hans-Carl-von-Carlowitz-Platz 2, 85354 Freising, Germany

^2^Chair of Wood Science, Holzforschung München, TUM School of Life Sciences, Technical University of Munich, Winzererstr. 45, 80797 Munich, Germany

^3^Institute for Advanced Study, Technical University of Munich, Lichtenbergstraße 2a, 85748 Garching, Germany

This file contains:

Figure S1

Figure S2

Figure S3

Figure S4
Figure S5

Table S1

Table S2

Table S3

**Supplemental Figures**


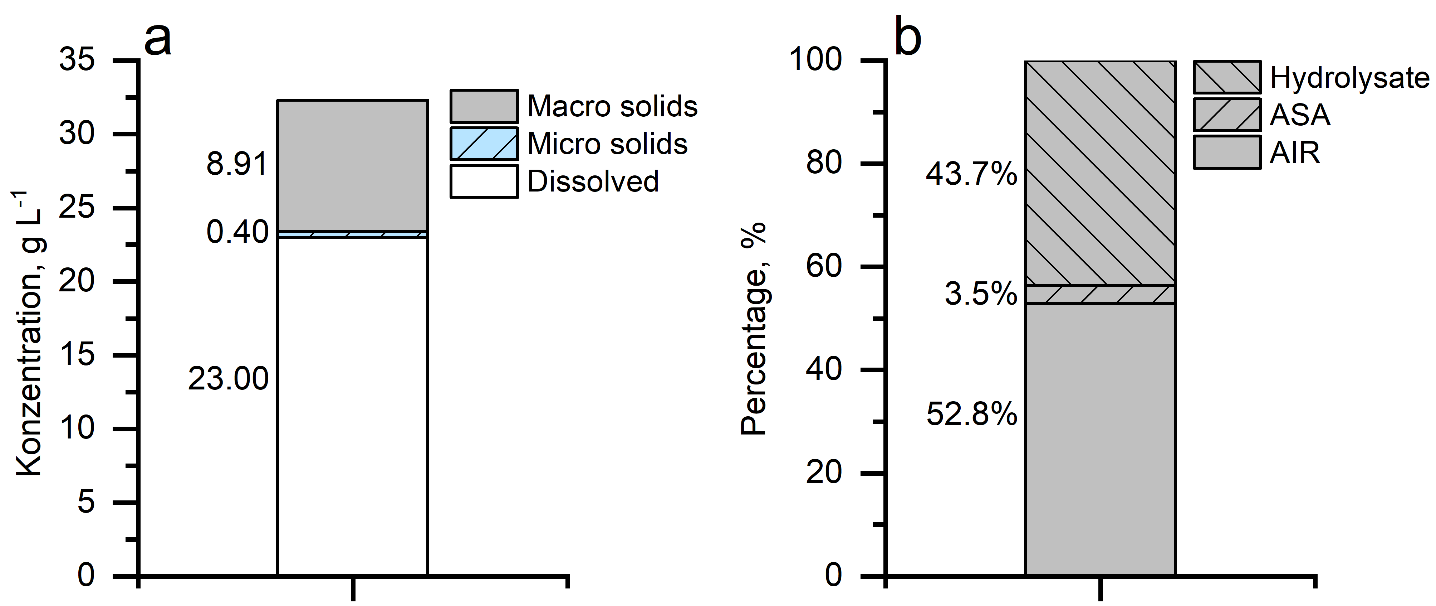


Fig. S1 Physico-chemical analysis of press water from Douglas-fir with bark (PWB). **a** Concentration of macro solids, micro solids and dissolved substances. **b** Mass percentage of hydrolysate, acid soluble aromatics, and acid insoluble residues of PWB solids after acid hydrolysis.


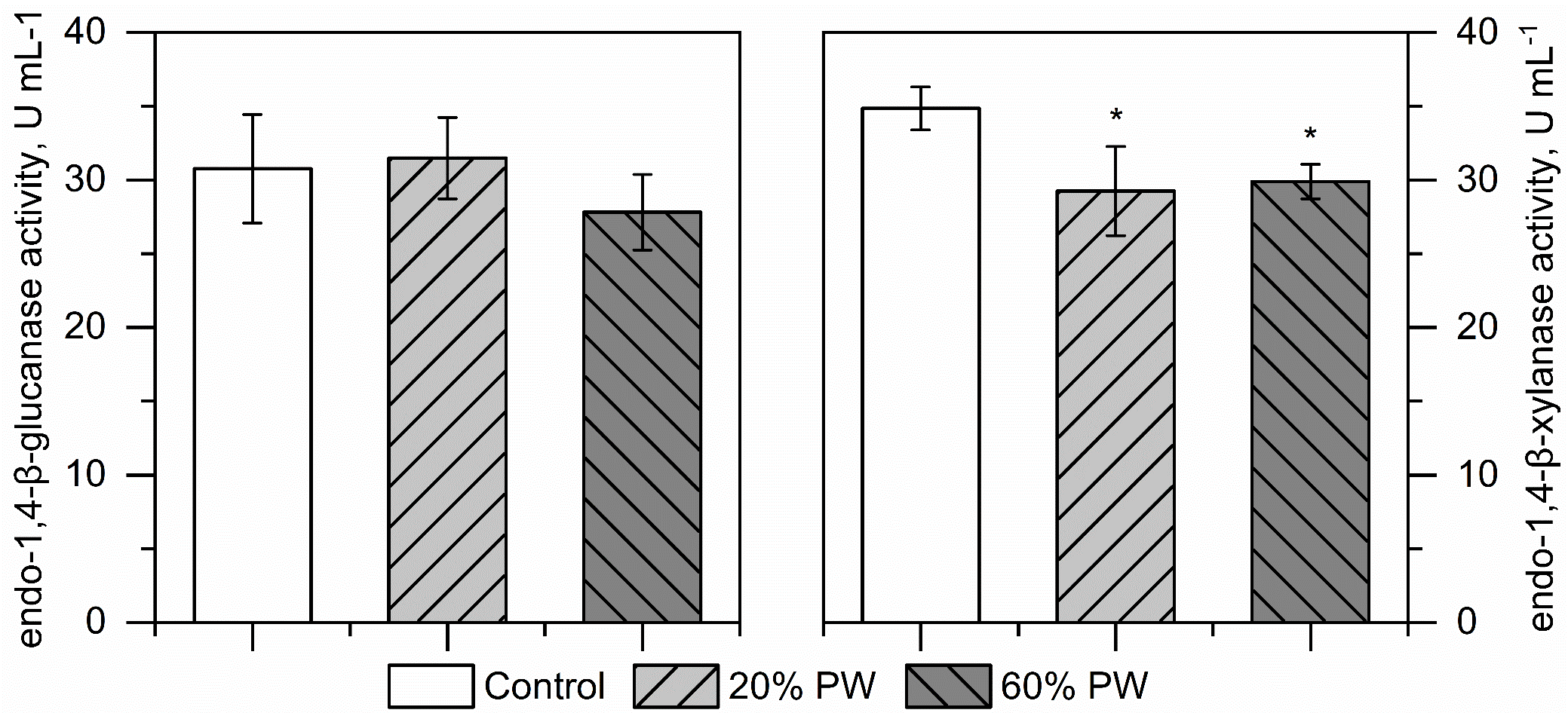


Fig. S2 Endoglucanase and endoxylanase activities of *T. reesei* RUT-C30 culture supernatants in the presence of increasing PW (% v/v) concentrations. Cultivation in MA medium with 1 % Avicel at 30 °C and 250 rpm. Significant differences (p>0.05) relative to the control are indicated by asterisks.


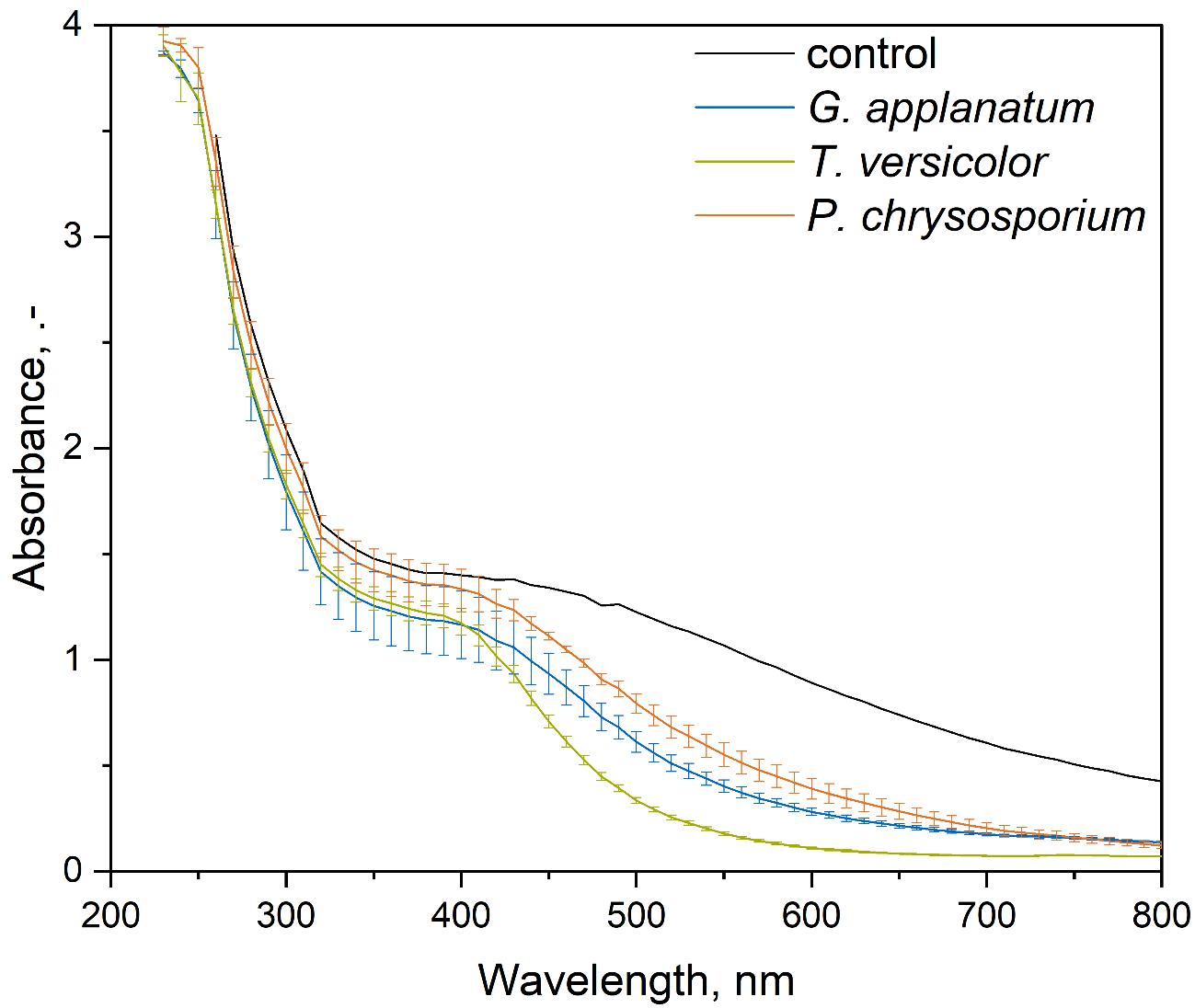


Fig. S3 Absorbance scan of the culture supernatants of *G. applanatum*, *T. versicolor*, and *P. chrysosporium* cultivated in 50% PW with potato dextrose yeast medium (PDY) in shaking flasks with 30 mL medium, at 100 rpm (50 mm throw), 28 °C. Absorbance in the range of 230 – 800 nm was measured in UV transparent cuvettes. Error bars indicate the standard deviation of biological triplicates. The control was not measured in replicates.


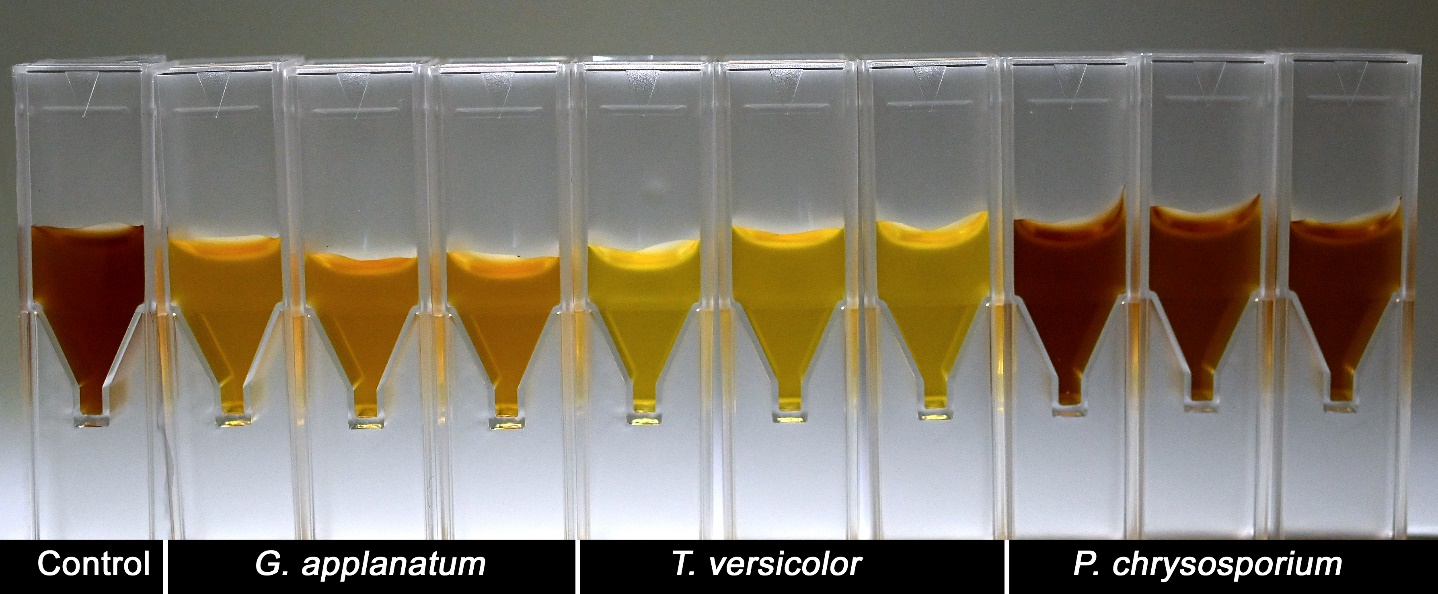


Fig. S4 Photograph of the culture supernatants of *G. applanatum*, *T. versicolor*, and *P. chrysosporium* cultivated in 50% PW with potato dextrose yeast medium (PDY) in shaking flasks with 30 mL medium, at 100 rpm (50 mm throw), 28 °C.


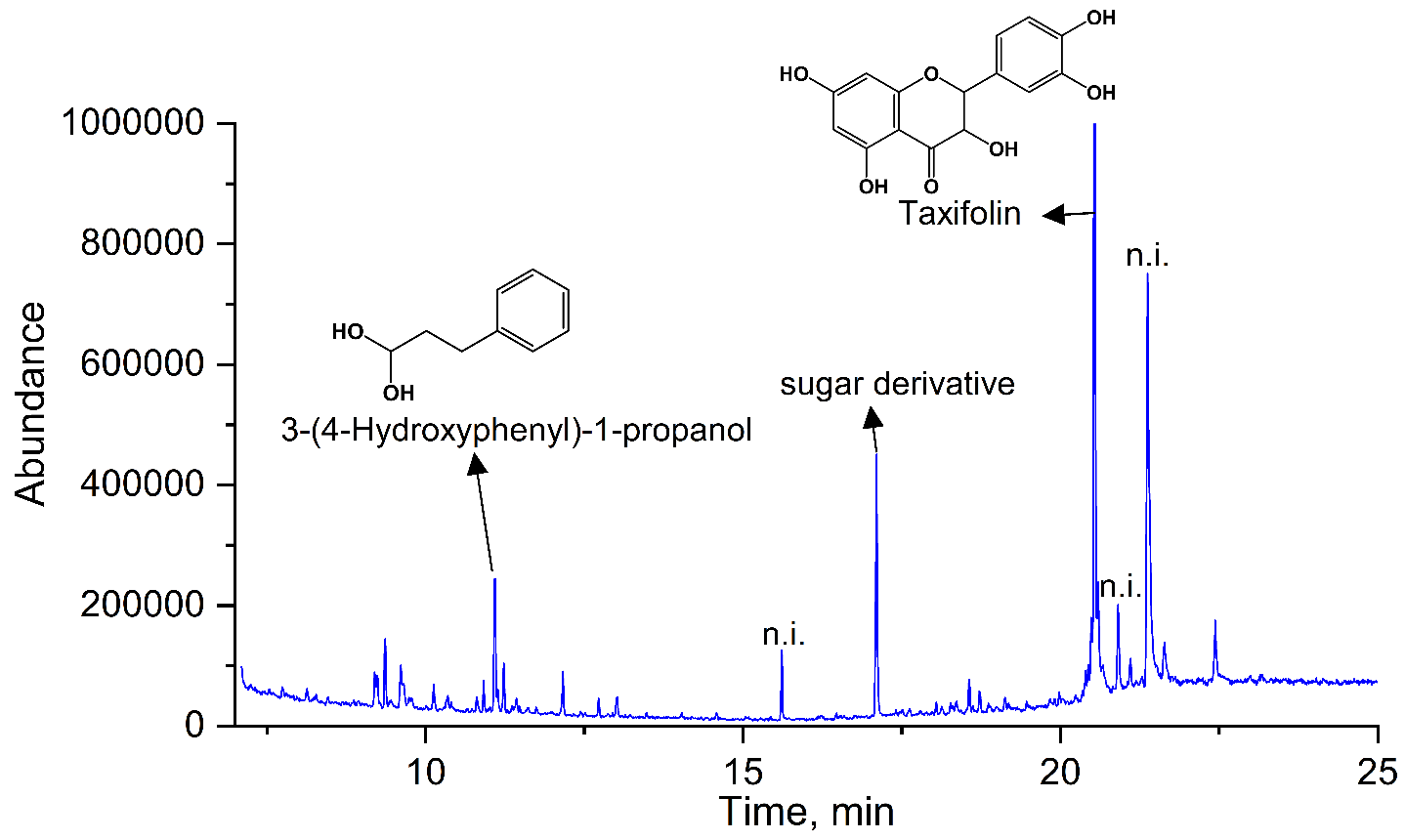


Fig. S5 Chromatogram of the GC/MS of the hydrophobic fraction of dissolved substances in the PW, concentrated with a C18ec SPE cartridge. Chemical formulas for 3-(4-Hydroxyphenyl)-1-propanol and taxifolin are showed. N.i., not identified.

**Supplementary Tables**

Table S1: Ion concentration in PW Douglas-fir with bark relative to Mandels-Andreotti medium (MA)

|  | **PW** | |  | **MA-medium** | |
| --- | --- | --- | --- | --- | --- |
|  | mg L^-1^ | mM |  | mM | covered by PW, % |
| **PO_4_^3-^** | 34.5 | 0.36 |  | 14.7 | 2.5 |
| **SO_4_^2-^** | 29.6 | 0.31 |  | 11.85 | 2.6 |
| **NH_4_^+^** | 4.78 | 0.26 |  | 21.19 | 1.3 |
| **NO_3_^-^** | < 5 | - |  | - | - |
| **Na^+^** | 50.8 | 2.21 |  | - | - |
| **Ca^2+^** | 133 | 3.32 |  | 2.72 | 122.0 |
| **Mg^2+^** | 40.1 | 1.65 |  | 1.22 | 135.2 |
| **K^+^** | 109 | 2.79 |  | 14.7 | 19.0 |
| *Trace elements* | | | | | |
| **Fe^2+^** | 49.3 | 0.88 |  | 0.02 | 4414.0 |
| **Mn^2+^** | 13.8 | 0.25 |  | 0.01 | 2511.9 |
| **Zn^2+^** | 1.34 | 0.02 |  | 0.005 | 409.8 |

Table S2: Compositional analysis of Douglas-fir wood chips (pressed and unpressed) expressed in % of the total weight

|  | Extractives | Lignin insoluble | Lignin soluble | Rha | Man | Ara | Gal | Xyl | Glu | Sum |
| --- | --- | --- | --- | --- | --- | --- | --- | --- | --- | --- |
|  | [%] | [%] | [%] | [%] | [%] | [%] | [%] | [%] | [%] | [%] |
| pressed | 1.36 | 27.6 | 0.6 | < 0,1 | 13.6 | 0.9 | 2.7 | 3.7 | 46.6 | 97.1 |
| not pressed | 1.53 | 27.3 | 0.7 | < 0,1 | 12.8 | 0.9 | 2.7 | 3.5 | 45.7 | 95.1 |

Table S3: Concentration of dissolved sugars in Douglas-fir PW with bark measured in a HPAEC-PAD.

| Sugars | Concentration |
| --- | --- |
|  | [g L^-1^] |
| Mannose | 0.012 ± 0.002 |
| Rhamnose | 0.023 ± 0.003 |
| Sucrose | 0.028 ± 0.002 |
| Arabinose | 0.054 ± 0.001 |
| Cellobiose | 0.063 ± 0.009 |
| Galactose | 0.066 ± 0.002 |
| Xylose | 0.112 ± 0.001 |
| Glucose | 1.827 ± 0.128 |
| Fructose | 2.318 ± 0.124 |
